# Impact of Tooth Preparation Techniques on Fracture Strength of Composite‐Restored Teeth: A Focus on Deep Margin Elevation With Sandblasting and EDTA

**DOI:** 10.1155/ijod/5514582

**Published:** 2026-11-03

**Authors:** Horieh Moosavi, Salehe Sekandari, Amin Shayesteh, Afshin Kordestani, Hossein Bagheri, Maryam Abrishamchi

**Affiliations:** ^1^ Department of Restorative Dentistry School of Dentistry Mashhad University of Medical Sciences Mashhad Iran mums.ac.ir; ^2^ School of Dentistry Mashhad University of Medical Sciences Mashhad Iran mums.ac.ir; ^3^ Dental Materials Research Center Mashhad University of Medical Sciences Mashhad Iran mums.ac.ir

**Keywords:** composite inlay, composite onlay, DME technique, EDTA, sandblast

## Abstract

**Objective:**

This study aimed to investigate the effect of dentin surface pretreatment with sandblasting and 17% ethylenediaminetetraacetic acid (EDTA) on the fracture strength of composite inlay and onlay restorations placed using deep margin elevation (DME).

**Materials and Methods:**

Sixty extracted human premolars with standardized mesio‐occluso‐distal (MOD) cavity preparations were randomly assigned to composite inlay or onlay groups (*n* = 30). Standardized cusp reduction (2 mm) was performed in the onlay group. Immediate dentin sealing (IDS) and DME were performed in all specimens. Each group was subdivided according to dentin pretreatment: control, sandblasting, or 17% EDTA (*n* = 10/subgroup). Semi‐direct composite restorations were fabricated and adhesively cemented. Following thermocycling, fracture strength was assessed using a universal testing machine, and fracture patterns were qualitatively evaluated. Data were analyzed using two‐way analysis of variance (ANOVA).

**Results:**

All 60 teeth were included in the final analysis. The mean fracture strength of onlay restorations was higher than that of inlay restorations across all surface treatment groups. A statistically significant difference between inlay and onlay restorations was observed in the EDTA (*p* = 0.012) and control (*p* = 0.018) groups, whereas the sandblasting group showed no significant difference (*p* = 0.107). Two‐way ANOVA revealed a significant main effect of restoration type on fracture strength (*p* < 0.001, partial *η* ^2^ = 0.216), while the main effects of surface treatment (*p* = 0.457, partial *η* ^2^ = 0.029) and the interaction between restoration type and surface treatment (*p* = 0.994, partial *η* ^2^ = 0.0002) were not statistically significant. Sandblasting demonstrated the highest mean fracture strength, followed by the control and EDTA groups, although these differences were not statistically significant. No significant differences were observed in fracture repairability patterns among groups.

**Conclusions:**

In each preparation, the fracture strength of the inlay group was lower than that of the onlay group. Furthermore, the highest resistance across the different preparations was observed for sandblasting, followed by the control, and finally EDTA, but the differences were not statistically significant.

## 1. Introduction

One of the main reasons for replacing posterior composite restorations is recurrent caries, which is due to the failure at the interface bond. This failure is due to shrinkage during polymerization, which leads to stress formation within the restoration and the creation of a gap at the margin. Several methods have been proposed to solve this problem, including placing small composite pieces, the indirect method, etc. In the indirect method, polymerization shrinkage occurs before cementation. In several studies, the advantages of this method have been mentioned, including higher strength, proper adaptation, and reduced microleakage [1]. Indirect restorations, including inlays, onlays, and endocrowns, offer improved anatomical contours [2, 3]. Studies have investigated the clinical survival rates of direct and indirect restorations in posterior and permanent teeth [4–6]. Indirect restorations appear to have a significantly lower average annual failure rate than direct restorations [5].

Due to the less vertical extension of the indirect restoration to the proximal floor in deep margin elevation (DME) teeth, complete seating of the restoration is possible in these areas, leading to better marginal adaptation. Therefore, the fracture strength is higher in DME teeth [7]. In the study by Zhang et al. [8], it was shown that the use of the DME method, regardless of the type of restorative material used, leads to increased fracture strength in root‐canal‐treated premolars.

Although indirect composite restorations exhibit improved mechanical properties and reduced polymerization shrinkage compared with direct restorations, the long‐term performance of these restorations remains highly dependent on the quality and durability of the adhesive interface. Degradation of the adhesive bond over time may compromise marginal integrity and ultimately affect the longevity and fracture resistance of the restored tooth–restoration complex. Therefore, various adhesion protocols and dentin surface pretreatment strategies have been proposed to optimize bonding performance and improve restoration durability [9, 10]. Additionally, the degradation of the adhesive bond over time significantly impacts the longevity of adhesive restorations [11]. Detecting defects at the tooth/restoration interface can serve as an early indicator of adhesive restoration failure, potentially jeopardizing the restoration’s durability [12]. Over the years, various adhesion protocols, alternative materials, and surface pretreatment modifications have been explored to enhance adhesion [13]. Chemical or mechanical dentin pretreatment methods can increase the surface roughness of the treated area, potentially improving the adhesive strength by enlarging the contact area between the dentin and the adhesive [14].

Among the dental pretreatments that enhance tooth cleaning, the sandblasting (air abrasion) method is notable for increasing surface roughness and adhesion [15]. The use of aluminum oxide in sandblasting further enhances tooth surface roughness, creating more favorable conditions for bonding [16]. Several studies have been conducted to evaluate the effectiveness of this treatment [16–18], which mentioned a higher bond strength.

Various materials have been used for the surface preparation of enamel and dentin, including phosphoric, maleic, nitric, citric, and ethylenediaminetetraacetic acids (EDTA). These acids are used to remove the smear layer and demineralize the underlying enamel and dentin [19]. EDTA is a molecule containing 4 carboxyl groups and can bind to calcium. It is widely used to dissolve dentin minerals without altering dentin proteins and prevents major changes in the fibrillar collagen structure of dentin [20]. Previous studies have reported that EDTA pretreatment may improve dentin conditioning by selectively removing the smear layer while preserving the collagen structure, potentially enhancing adhesive performance when used prior to self‐etch systems [20–22].

Since dentin pretreatment procedures may alter the characteristics of the adhesive interface and the integrity of the tooth–restoration complex, different surface treatment strategies performed before immediate dentin sealing (IDS) and DME could potentially influence the fracture resistance of restored teeth. However, evidence regarding the effect of such dentin pretreatment protocols on the mechanical behavior of teeth restored with indirect composite restorations remains limited. Despite the growing evidence on adhesive strategies and indirect restorative techniques, there is limited information regarding how different dentin surface pretreatments in DME influence the fracture strength of composite restorations. Since fracture resistance is a key determinant of the clinical longevity of posterior composite restorations, understanding the effect of such pretreatments is essential. Therefore, the aim of this in vitro pilot study was to investigate the effect of dentin surface pretreatment with sandblasting and EDTA on the fracture strength and fracture pattern of teeth restored with indirect composite inlay and onlay restorations in the presence of DME and IDS. The null hypothesis was that dentin surface pretreatment using sandblasting or EDTA would not affect the fracture strength or fracture pattern of restored teeth.

## 2. Materials and Method

### 2.1. Study Design

The present research is a laboratory study investigating the effect of different tooth preparations in the DME technique, including sandblasting and EDTA, on the fracture strength of composite‐restored teeth. Fracture patterns were additionally assessed as a qualitative secondary observation. Throughout this manuscript, the term “fracture strength” refers to the maximum load at which specimen failure occurred under compressive loading in a universal testing machine. The study was conducted at the Mashhad University of Medical Sciences, School of Dentistry from August 2022 to September 2023. Approval for the study was obtained from the ethics committee of Mashhad University of Medical Sciences, School of Dentistry, with Project Numbers 4010376 and 4010416 (Ethic Codes IR.MUMS.DENTISTRY.REC.1401.05 and IR.MUMS.DENTISTRY.REC.1401.058). Also, this in vitro study was designed and reported in accordance with the Checklist for Reporting In‐vitro Studies (CRIS) guidelines (Table S1). In this study, extracted teeth that could not be maintained due to periodontal disease and teeth that needed to be extracted due to a lack of space in orthodontic treatments were used. Also, as this was an in vitro pilot study, no formal sample size calculation was performed. However, the sample size per group was chosen based on previously published similar studies in restorative dentistry and feasibility constraints. The findings should therefore be interpreted with the consideration of the exploratory nature of the study. Thus, the inclusion criteria were (a) extracted human premolar teeth and (b) intact teeth without caries or restorations. The exclusion criteria were as below: (a) cracked teeth; (b) enamel defects; (c) teeth with caries; and (d) previously restored teeth.

### 2.2. Intervention

Sixty permanent human premolars without previous restoration and carious lesions were selected for the study. Teeth presenting caries, cracks, enamel defects, or structural defects were excluded from the study. The dental plaques, bone debris, and soft tissue adhering to the root surface were removed with a scaler. The teeth were placed in a 0.5% thymol solution for 48 h and stored in a 0.9% saline solution at room temperature after rinsing until the start of the experiment. All specimens were stored under identical conditions and tested within 2 months of extraction, which is consistent with storage periods commonly reported in previous in vitro restorative studies and with the recommendations provided in ISO/TS 11405 for testing adhesion to the tooth structure [23]. A digital caliper carefully measured the teeth, and the teeth of each group were tried to have similar dimensions to the other groups. The samples were selected in the same dimension range of buccolingual, mesiodistal, and occluso‐cervical, with a standard deviation of 10% ± from the mean. The teeth were mounted in the center of a custom‐made generator in Acropars 200 methacrylate resin (Marlic Medical Industries Co., Tehran, Iran). All 60 teeth received standardized mesio‐occluso‐distal (MOD) cavity preparations using a high‐speed handpiece and diamond burs under water cooling. The internal line angles were rounded to reduce the stress concentration, and no sharp internal angles were present. The cavosurface margins were prepared with a butt‐joint configuration at 90° without beveling. Axial walls were prepared with a slight occlusal convergence to facilitate restoration insertion. The depth of the proximal boxes was extended to 2 mm below the cementoenamel junction (CEJ) (Figure 1). All preparations were standardized in terms of geometry and dimensions to ensure consistency among the specimens. Specimens were then randomly allocated to the experimental groups using a computer‐generated randomization sequence. In the onlay groups, standardized reduction of the buccal and lingual cusps was performed to a depth of 2 mm from the cusp tip, and the reduction depth was verified using a digital caliper. In these stages, the Black Peral turbine (Bein Air Dental SA, Bienne, Switzerland) and the milling with a diameter of 1.2 mm and a length of 8 mm Diamond Bur Cylinder L 837 (Verdent sp. z. o.o., Łódź, Poland) were used. Immediately after the preparation of the etched surfaces, all specimens underwent DME and IDS treatment. IDS was performed immediately after tooth preparation using a two‐step self‐etch adhesive system (Clearfil Liner Bond F; Kuraray Noritake Dental Inc., Tokyo, Japan) according to the manufacturer’s instructions. After the application of the primer and bonding agent, a thin hydrophobic resin layer was applied using a flowable composite (Clearfil AP‐X Flow) to ensure complete sealing of the dentin surface. This layer was then light‐cured for 20 s to complete polymerization and stabilize the sealed dentin surface before further restorative procedures. However, different surface preparations were performed on the dentin before applying the adhesive in different subgroups.

**Figure 1:**
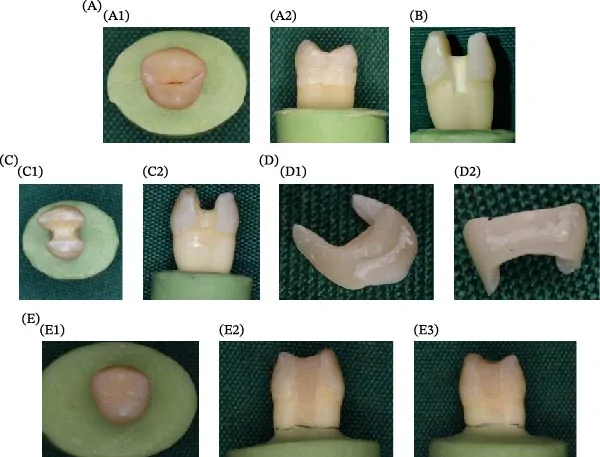
Steps of inlay group preparations. (A) Selected teeth mounted before grinding. (B) Ground tooth. (C) Tooth view after DME and IDS. (D) Image of the inlay before cementation. (E) View of the teeth after cementation of the inlay and polishing.

#### 2.2.1. Group I: Composite Inlay Restorations (Figure 1)

• Group I‐1 (*n* = 10): performing IDS only using a self‐etch adhesive (Clearfil Liner Bond F; Kuraray Noritake Dental Inc., Tokyo, Japan).
• Group I‐2 (*n* = 10): sandblasting with 50 microns aluminum oxide microblaster powder particles (Westcode, Foshan Snyder Medical Equipment Co., Ltd) with a time of 2 s and pressure of 2 bar from 1 cm from the surface of the occlusal surface, then performing the IDS using the self‐etch adhesive (Clearfil Liner Bond F).
• Group I‐3 (*n* = 10): 60‐s application of 17% EDTA solution (Doradent DORA.EDTA‐S, Dorsan Darman Co., Tehran, Iran) equivalent to 0.1 molar, washing, and performing IDS using the self‐etch adhesive (Clearfil Liner Bond F).

#### 2.2.2. Group O: Composite Onlay Restorations (Figure 2)

• Group O‐1 (*n* = 10): performing IDS only using a self‐etch adhesive (Clearfil Liner Bond F; Kuraray Noritake Dental Inc., Tokyo, Japan).
• Group O‐2 (*n* = 10): sandblasting with 50 microns aluminum oxide microblaster powder particles (Westcode, Foshan Snyder Medical Equipment Co., Ltd) with a time of 2 s and pressure of 2 bar from 1 cm from the surface of the occlusal surface, then performing the IDS using the self‐etch adhesive (Clearfil Liner Bond F).
• Group O‐3 (*n* = 10): 60‐s application of 17% EDTA solution (Doradent DORA.EDTA‐S, Dorsan Darman Co., Tehran, Iran) equivalent to 0.1 molar, washing, and performing IDS using the self‐etch adhesive (Clearfil Liner Bond F).

After performing IDS in all groups, DME was performed using a Clearfil AP‐X microhybrid composite layer (Kuraray Noritake Dental Inc., Tokyo, Japan) shade A2 according to the manufacturer’s instructions up to the CEJ. After DME, the remaining dentin surfaces were sealed with a thin layer of Clearfil AP‐X Esthetics Flow composite (Kuraray Noritake Dental Inc., Tokyo, Japan) shade XW (Figures 1 and 2). The light‐curing time for the composite used for DME was 20 s (per side) according to the manufacturer’s instructions, and 10 s for the specified bonding agent according to the manufacturer’s instructions. To remove the air‐inhibited layer, all teeth were treated with glycerin gel (Kimia Darou Sepehr., Eshtehard, Iran) after DME and IDS and were light‐cured again for 20 s. All light‐curing procedures were performed using the iLed Plus light‐curing unit (Guilin Woodpecker Medical Instrument Co., China) with a minimum power of 580 mW/cm^2^. After preparation and DME, the teeth were stored in water in an incubator until the inlays and onlays were ready.

**Figure 2:**
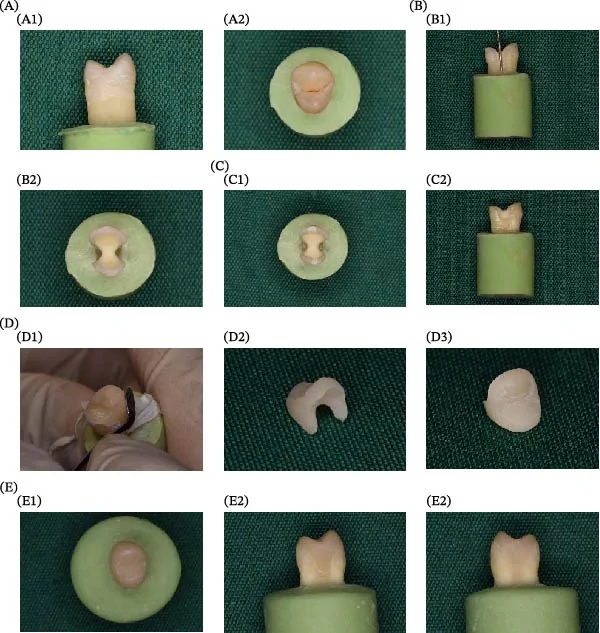
Steps of onlay group preparations. (A) Tooth mounted in acrylic before trimming. (B) Teeth after MOD cavity preparation and cusp reduction (onlay preparation). (C) Teeth after DME and IDS. (D) Stages of composite onlay fabrication. (E) Teeth after cementation of the composite onlay.

The inlay restorations were fabricated using a microhybrid composite resin system (CLEARFIL AP‐X microhybrid composite, shade A2; Kuraray Noritake Dental Inc., Tokyo, Japan) through a semi‐direct technique. A Teflon strip was adapted over the cavity, and the composite resin was incrementally placed and light‐cured to form the inlay restoration.

The onlay restorations were fabricated using a microhybrid composite resin system (CLEARFIL AP‐X, shade A2; Kuraray Noritake Dental Inc., Tokyo, Japan) through a semi‐direct technique. A Teflon tape was adapted over the cavity, and the composite resin was incrementally layered and light‐cured to form the onlay restorations.

For both restoration types, the restorations were carefully removed and subjected to extraoral postcuring. Prior to cementation, restorations were evaluated for seating and marginal adaptation, and minor adjustments were performed when necessary to ensure a proper fit. No specimens were excluded due to an inadequate fit. Final polymerization was completed in a laboratory light‐curing unit (Targis Power Polymerization Unit, Ivoclar Vivadent AG, Schaan, Liechtenstein) at 104°C for 25 min. The same postcuring protocol (104°C for 25 min) was applied to both inlay and onlay restorations using the same polymerization unit to ensure procedural standardization (Figures 1 and 2). Both restorative procedures were performed using identical materials and polymerization protocols; the only difference between groups was the cavity design (inlay vs. onlay).

Before cementation, the internal surfaces of the restorations and the tooth surfaces treated with IDS and DME were air‐abraded using an aluminum oxide microblaster for 10 s at a pressure of 2 bar and a working distance of 1 cm from the surface. After preparation, they were rinsed and dried, followed by etching the tooth surfaces for 30 s and the internal surfaces of the restorations for 10 s with 37% phosphoric acid (Doradent Dorsan Etch, Dorsan Darman Co., Tehran, Iran). After preparation, the internal surfaces of the composite restorations were air‐abraded and subsequently treated with a silane‐containing porcelain primer (Bis‐Silane Porcelain Primer, Bisco Inc., Illinois, USA) according to the manufacturer’s instructions. Silane application was performed because air abrasion may expose silica‐containing filler particles within the indirect composite substrate, potentially allowing chemical interaction between silane molecules and the exposed fillers. Previous studies have suggested that silanization following air abrasion may improve bonding to indirect composite surfaces by enhancing chemical coupling and surface wettability [24, 25]. Nevertheless, the additional benefit of silane application to indirect composite substrates remains controversial and may depend on the amount of exposed filler particles after the surface treatment. The tooth surfaces were also prepared using Clearfil Liner Bond F primer and bond (Kuraray Noritake Dental Inc., Tokyo, Japan) without curing, following the provided instructions.

Finally, the prepared composite restorations were cemented to the teeth using a dual‐cure self‐adhesive cement (Clearfil SA Luting dual‐cure self‐adhesive cement, Kuraray Noritake Dental Inc., Tokyo, Japan) for each group. Postcementation light curing was performed from three directions (mesial, distal, and occlusal), with each direction receiving 20 s of exposure. Tooth isolation was maintained for 5 min to allow for chemical setting, after which the samples were placed in an incubator for 24 h to ensure complete polymerization (Figures 1 and 2). Thermocycling was conducted on all teeth using a Thermocycle Machine (Nemove Co., Tehran, Iran) under the following conditions: 10,000 temperature cycles, with each cycle consisting of 30 s at each temperature with a 15‐s transition time, in a temperature range of 10–60°C. Fracture strength testing was performed immediately after completion of the thermocycling procedure.

To evaluate fracture strength, the teeth were tested in a Universal testing machine (Santam Co., Tehran, Iran) with a measurable load cell and a speed of 0.5 mm/min until fracture occurred. The lever of the machine was positioned on the tooth at a 90° angle in the middle of the occlusal surface (Figure 3A,B). After fracture testing, fracture patterns were qualitatively assessed as favorable (repairable) or unfavorable (nonrepairable) using a stereomicroscope (Zeiss, Jena, Germany) at ×20 magnification by two independent observers who were blinded to the experimental group allocation. The final classification was established by consensus between the two observers. However, inter‐rater reliability was not formally quantified using statistical measures such as Cohen’s kappa.

**Figure 3:**
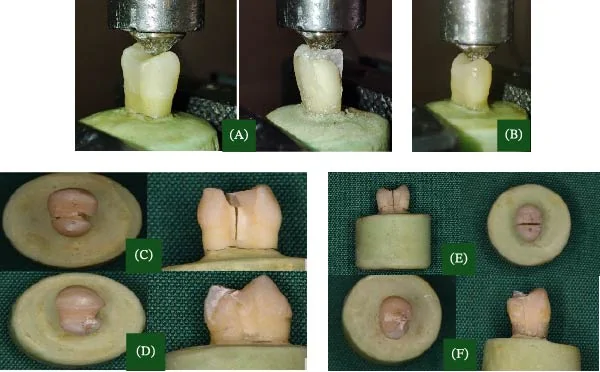
The fracture strength test and types of fractures in groups. (A, B) A sample undergoing a fracture strength test in the inlay and onlay groups, respectively. (C, E) Examples of unfavorable fracture in the inlay and onlay groups, respectively. (D, F) Examples of favorable fracture in the inlay and onlay groups, respectively.

### 2.3. Statistical Analysis

Given that the study was a pilot study, a sample size of 10 in each group was considered. Mean and standard deviation indices were used to describe the data. Data distribution was assessed using the Shapiro–Wilk test. A two‐way analysis of variance (ANOVA) was performed to evaluate the effects of surface treatment, restoration type, and their interaction on fracture strength. Independent‐samples *t*‐tests were used for pairwise comparisons of fracture strength between inlay and onlay restorations within each surface treatment group. Fracture repairability patterns (favorable vs. unfavorable fractures) were analyzed using Fisher’s exact test. The software used was SPSS Version 20. The significance level in the statistical tests was 0.05.

## 3. Results

In the present study, 60 teeth were investigated. Groups are presented in Table 1. No teeth were missed, and all were included in the final analysis. The descriptive information on the fracture strength of each experimental group is presented in Table 1. The mean fracture strength of the onlay group was higher than that of the inlay group in each surface treatment. Representative photographs of the fracture strength testing procedure and examples of favorable and unfavorable fracture patterns are presented in Figure 3. Fracture patterns were qualitatively categorized as favorable (repairable) or unfavorable (nonrepairable), with no statistically significant differences among groups (*p* > 0.05). The distribution of fracture patterns according to surface treatment in the inlay and onlay groups is presented in Table 2.

**Table 1:** Descriptive statistics of study groups.

Group	Restoration type	*N*	Mean (±SD)	Range (min–max)	5% trimmed mean
EDTA	Inlay	10	843.50 (±332.09)	264.00–1267.00	852.17
	Onlay	10	1214.30 (±252.63)	749.00–1572.00	1220.28
Sandblasting	Inlay	10	991.80 (±380.99)	542.00–1720.00	976.33
	Onlay	10	1338.10 (±521.39)	491.00–2043.00	1346.00
Control	Inlay	10	956.30 (±267.64)	616.00–1309.00	955.61
	Onlay	10	1321.70 (±352.58)	841.00–2107.00	1304.78

*Note:* Data are presented as mean (±SD). The reported unit is Newton. The 5% trimmed mean is presented as a robust descriptive statistic that reduces the influence of extreme values by excluding the highest and lowest 5% of observations. Since no substantial differences were observed between the trimmed means and conventional means, standard means were used for all inferential statistical analyses.

**Table 2:** Distribution of fracture patterns according to surface treatment in the inlay and onlay groups.

Restoration type	Group (surface treatment)	Favorable *n* (%)	Unfavorable *n* (%)	*p*‐Value ^∗^
Inlay	Control	6 (60)	4 (40)	0.189
	Sandblasting	8 (80)	2 (20)	
	EDTA	4 (40)	6 (60)	
Onlay	Control	2 (20)	8 (80)	0.535
	Sandblasting	1 (10)	9 (90)	
	EDTA	3 (30)	7 (70)	

*Note: p*‐Values were calculated using Fisher’s exact test. Favorable fractures were considered repairable, whereas unfavorable fractures were considered nonrepairable.

^∗^Data are presented as *n* (%).

A two‐way ANOVA was performed to assess the effects of surface treatment, restoration type, and their interaction on the fracture strength. The analysis revealed a statistically significant main effect of restoration type (*F* = 14.889, *p* < 0.001, partial *η* ^2^ = 0.216), indicating a large effect size and suggesting that restoration type significantly influenced fracture strength. The main effect of surface treatment was not statistically significant (*F* = 0.796, *p* = 0.457, partial *η* ^2^ = 0.029), indicating only a small effect size. The interaction effect between surface treatment and restoration type was also not significant (*F* = 0.006, *p* = 0.994, partial *η* ^2^ = 0.0002), indicating a negligible interaction effect. Overall, the model explained 23.4% of the variance in the fracture strength (*R* ^2^ = 0.234).

Figure 4 presents the estimated marginal means of fracture strength for inlay and onlay restorations across different surface treatment groups. Onlay restorations consistently demonstrated higher fracture strength than inlays across all surface treatment groups. Sandblasting showed the highest mean values, followed by control and EDTA; however, these differences were not statistically significant.

**Figure 4:**
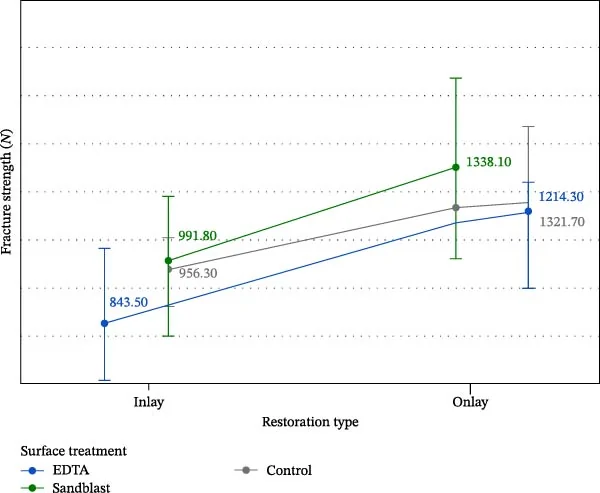
Estimated marginal means of fracture strength (*N*) for inlay and onlay restorations across different surface treatment groups. Error bars represent standard deviations.

Pairwise comparisons of fracture strength between inlay and onlay restorations within each surface treatment are presented in Table 3. These comparisons showed higher fracture strength in onlay restorations across all subgroups.

**Table 3:** Pairwise comparisons of fracture strength between inlay and onlay restorations within each surface treatment.

Group	Mean difference (*N*)	Std. error difference	95% CI of difference	*t*‐Value (df)	*p*‐Value
EDTA	−370.80	131.95	−648.01 to −93.59	−2.810 (18)	0.012
Sandblasting	−346.30	204.21	−775.32 to 82.72	−1.696 (18)	0.107
Control	−365.40	139.98	−659.49 to −71.31	−2.610 (18)	0.018

*Note:* These comparisons are presented for descriptive purposes; the primary analysis was performed using two‐way ANOVA. Mean differences were calculated as inlay–onlay fracture strength values. A *p*‐value <0.05 was considered statistically significant.

## 4. Discussion

Tooth‐colored restorations are increasingly popular due to esthetic reasons and concerns about mercury in amalgam. Direct composite restorations have limitations, particularly in larger cavities [2, 26]. It is possible to construct restorations with a better anatomical contour and more precise contact points than direct restorations using the indirect method [2].

We aimed to investigate the effect of different tooth preparations in the DME technique, including sandblasting and EDTA, on the fracture strength of composite‐restored teeth and their repairability after fracture strength testing. The rationale for using surface pretreatments in combination with a mild self‐etch adhesive is based on the limited demineralization capacity of self‐etch systems. While self‐etch adhesives partially interact with the smear layer, additional surface modifications such as sandblasting or EDTA application may enhance surface energy, remove loosely bound smear layer components, and improve resin infiltration without excessively compromising the dentin substrate. Therefore, the present study evaluated whether adjunctive mechanical (sandblasting) or chemical (EDTA) pretreatments could optimize the bonding performance of a mild self‐etch adhesive in DME substrates. This approach reflects a clinically relevant scenario where additional dentin conditioning is performed prior to the application of self‐etch systems to potentially enhance bonding performance in challenging substrates. In this regard, in vitro studies demonstrating the bonding ability or inability of a restorative material are important because they can predict the clinical behavior, durability, and long‐term success of that material. Another reason for the importance of such in vitro studies is that they can be performed quickly, easily, on a large number of samples, and by changing only one variable at a time [17]. Regarding restoration design, onlay restorations demonstrated higher fracture strength than inlay restorations across all experimental conditions, with statistically significant differences observed in the EDTA and control groups. This finding may be attributed to cusp coverage in onlay restorations, which potentially contributes to improved stress distribution and reinforcement of the weakened tooth structure. Regarding surface treatment, sandblasting demonstrated the highest mean fracture strength, followed by the control and EDTA groups; however, no statistically significant differences were observed among the surface treatment protocols. This finding suggests that under the present experimental conditions, adjunctive dentin pretreatment did not significantly influence fracture resistance. Because relatively few studies have investigated fracture strength following dentin pretreatment before DME, the available literature cited below predominantly consists of bond strength studies. These investigations are discussed to provide mechanistic insight into the potential influence of dentin pretreatment on the adhesive interface rather than as direct comparators to the fracture strength outcomes of the present study.

Clearfil Liner Bond F is a mild two‐step self‐etch adhesive system with a low pH (~2), which enables partial demineralization of the smear layer while maintaining residual hydroxyapatite for chemical interaction. The adhesive contains functional monomers such as 10‐MDP and HEMA, which contribute to dentin wettability and chemical bonding with calcium, potentially improving bonding durability. This adhesive strategy preserves part of the smear layer and facilitates monomer penetration into the underlying dentin, resulting in the formation of a thin hybrid layer [20]. Air abrasion has been shown to modify dentin surface morphology by increasing surface roughness and partially removing the smear layer, thereby increasing the available surface area for micromechanical interlocking with adhesive systems [27]. The results of the present study showed that the highest fracture strength was related to the sandblasted group, but no statistically significant difference was found in the mean fracture strength between the EDTA and sandblasting pretreatment methods compared to conventional methods. Although fracture patterns were qualitatively assessed as favorable or unfavorable, no statistically significant differences were observed among the experimental groups. An additional methodological aspect that should be considered when interpreting the findings is that all restorations underwent a standardized air‐abrasion procedure immediately before cementation. Consequently, specimens assigned to the sandblasting pretreatment groups received two sequential air‐abrasion procedures: one before IDS as the experimental intervention and another before cementation as part of the standardized luting protocol. Repeated air abrasion may have cumulatively modified the dentin and IDS‐treated surfaces by increasing surface roughness, altering the smear layer, and enhancing micromechanical retention. Therefore, the numerically higher fracture strength observed in the sandblasting groups may not be exclusively attributed to the initial dentin pretreatment but could also reflect the combined influence of both air‐abrasion procedures. Although no statistically significant differences were detected, this potential cumulative effect should be considered when interpreting the findings and should be controlled in future studies designed to isolate the specific contribution of pretreatment air abrasion.

Soares et al. [22] investigated the influence of different surface treatments on the bond strength of self‐etch adhesive systems. Although bond strength and fracture strength represent different biomechanical outcomes and cannot be directly compared, their findings provide mechanistic insight into how dentin surface pretreatments may influence adhesive interface quality. In their study, aluminum oxide sandblasting reduced bond strength, whereas in the present study, sandblasting was associated with numerically higher fracture strength, although the difference was not statistically significant. These differences may reflect the distinct mechanical outcomes evaluated and differences in the study design. Differences in substrate (bovine versus human dentin), specimen preparation, and mechanical testing methods further limit direct comparison between the two studies. Schilke et al. [28] demonstrated that dentinal tubule density is significantly higher in human dentin than in bovine dentin, which may partly explain the differences in adhesive behavior reported among studies.

A study in 2015 [16] investigated the effect of aluminum oxide powder air abrasion surface preparation. Also, another study [29] in 2008 investigated the effect of Er:YAG laser and air‐abrasion preparation methods on the microtensile bond strength of composite to dentin and enamel. Because these studies [16, 29] evaluated microtensile bond strength rather than fracture strength, they are cited only to provide mechanistic evidence regarding the influence of dentin pretreatment on adhesive performance and should not be interpreted as direct comparisons with the present fracture strength findings.

Luque‐Martinez et al. [21] conducted a randomized clinical trial evaluating the effect of EDTA pretreatment before the application of a one‐step self‐etch adhesive in noncarious cervical lesions. They reported a significantly higher restoration retention rate in the EDTA group after 18 months, suggesting that EDTA conditioning may improve the long‐term clinical durability of the adhesive interface. However, this clinical study investigated cervical direct composite restorations bonded with a one‐step self‐etch adhesive, which differs substantially from the present in vitro study involving indirect composite inlay/onlay restorations restored with IDS, DME, and a two‐step self‐etch adhesive system. Therefore, this study is cited to provide indirect clinical support for the potential influence of EDTA on adhesive performance rather than as a direct comparison with the present fracture strength findings. A 2020 study by Sinjari et al. [30] investigated the in vitro effect of sandblasting as an enamel pretreatment on bond strength. This study divided 32 bovine teeth into two groups of 16. In the test group, unlike the control group, sandblasting with 50‐μm aluminum oxide particles was performed before bonding. A scanning electron microscope (SEM) was used to evaluate the fracture topography. Statistical results showed a significant difference between the two groups, with higher tensile and fracture strength in the test group (with sandblasting). Since they evaluated tensile bond strength rather than fracture resistance, their findings are presented only as mechanistic evidence supporting the potential influence of air abrasion on adhesive performance. Differences in substrate, testing methodology, and air‐abrasion protocol further preclude direct comparison with the present study. Also, a study by Vanini and D’Arcangelo, on the effectiveness of various surface preparations on the adhesive properties of indirect composites, showed that sandblasting with aluminum oxide particles is the primary factor in enhancing adhesion [31].

In examining the mode of failure of the samples after the strength test, no statistically significant difference was found between the groups according to the type of preparation; however, due to its importance in clinical decision‐making, further research should be conducted in future studies. Although the EDTA group demonstrated a greater number of favorable fractures compared with the sandblasting group, this difference was not statistically significant.

The lack of difference in restorability between our study groups, despite identical preparation types, aligns with the findings of Mugri et al. [32] Their 2021 systematic review indicated that preparation design, rather than DME, significantly affects restorability (*p* < 0.001) and fracture type (*p* < 0.001). Like our study, they observed no significant difference in the fracture type with consistent preparation designs. Our study primarily aimed to evaluate the influence of different dentin pretreatment strategies on fracture strength. From a biomechanical perspective, improved bonding may contribute to a more integrated tooth–restoration complex and potentially influence fracture resistance. This concept has been supported by bonded indirect restorations, where resin cement facilitates stress distribution to the remaining tooth structure [33]. However, long‐term functional and parafunctional forces may compromise bond durability over time. This is supported by the example of bonded all‐ceramic crowns, where polymerization of the resin cement creates a strong union between the restoration and the tooth. This union formation facilitates force transfer to the underlying tooth structure during function. However, while properly bonded crowns initially exhibit high fracture strength, functional and parafunctional forces, combined with the properties of dentin, can compromise bond strength over time, potentially leading to restoration fracture [34].

Another methodological aspect that should be considered is the application of a silane‐containing primer to the air‐abraded indirect composite surfaces before cementation. Although silane coupling agents are well established for silica‐based ceramics, their effectiveness on indirect resin composites is less predictable and largely depends on the amount of exposed inorganic filler particles following surface treatment. Previous studies have demonstrated that silanization after air abrasion may enhance bonding to indirect composite substrates; however, other investigations have reported limited or no additional benefit [24, 25]. Therefore, while silane application was included in the present study based on available evidence and manufacturer recommendations, its specific contribution to the composite‐cement interface could not be isolated. Consequently, silane application should be considered an additional procedural variable when interpreting and reproducing the present findings.

This study has several strengths. Unlike previous studies that evaluated DME or dentin pretreatments separately, the present study assessed their combined influence on the fracture strength of composite inlay and onlay restorations. Clinically relevant procedures, including IDS, DME, semi‐direct restoration fabrication, postcuring, and thermocycling, were incorporated to better simulate restorative conditions. However, some limitations should be acknowledged. As a pilot in vitro study, the sample size was limited, and no a priori power calculation was performed. In addition, inter‐rater reliability for fracture mode classification was not formally quantified using the Cohen’s kappa. Furthermore, no statistical power estimate was performed for the surface‐treatment comparison, which represented the primary research question of the present study. Therefore, the null finding for the effect of surface treatment (*p* = 0.457) should be interpreted with caution, as the study may have been underpowered to detect clinically meaningful differences among the pretreatment groups. These limitations should be considered when interpreting the exploratory and hypothesis‐generating nature of the present findings. Based on the observed variance and the effect of restoration type (inlay versus onlay) identified in this pilot study, an exploratory post hoc sample size estimation indicated that ~17 specimens per group would be required for a future confirmatory study to achieve 80% statistical power at a two‐sided significance level of 0.05. Because this estimate was specifically based on the observed inlay–onlay comparison and was not intended to determine the sample size required to detect differences among surface‐treatment groups, it should be interpreted cautiously and used only to guide the design of adequately powered future studies. Another factor that should be considered is the output intensity of the light‐curing unit used in this study. Although all specimens were polymerized using the same standardized curing protocol, the minimum output intensity of the curing unit (580 mW/cm^2^) may have influenced the degree of conversion of the adhesive, DME composite, and resin cement layers. Consequently, variations in polymerization efficiency could have affected the mechanical properties of the bonded complex and contributed to variability in the fracture strength outcomes. Another limitation of the present study is that the specimens were mounted in acrylic resin without the simulation of the periodontal ligament. Since the stiffness of the mounting medium may influence the stress distribution during loading, the absolute fracture strength values obtained in this in vitro model should be interpreted with caution. Although thermocycling was used to simulate aging, mechanical loading and periodontal ligament simulation were not included. In addition, only one EDTA application protocol (60 s) was evaluated, and the standardized air‐abrasion step before cementation may have influenced surface characteristics in the sandblasting pretreatment groups. Therefore, future studies should incorporate more comprehensive oral environment simulation, including mechanical and chemical aging, in addition to thermocycling. Furthermore, larger laboratory investigations and clinical studies are warranted to confirm the present findings.

## 5. Conclusions

Considering the limitations of this study, our results show that the fracture strength of the inlay group was lower than that of the onlay group in each pretreatment. Furthermore, the highest resistance across the different preparations was observed for sandblasting, followed by the control, and finally EDTA, but the differences were not statistically significant.

## Author Contributions

**Horieh Moosavi**: conceptualization, methodology, investigation, writing – original draft. **Amin Shayesteh**: methodology, investigation, formal analysis, writing – review and editing. **Afshin Kordestani**: conceptualization,methodology, formal analysis, writing – review and editing. **Hossein Bagheri**: supervision, project administration, writing – review and editing. **Salehe Sekandari**: supervision, writing – review and editing. **Maryam Abrishamchi**: supervision, project administration, formal analysis, writing – review and editing.

## Funding

This work was supported by the Mashhad University of Medical Sciences, School of Dentistry (Grants 4010376 and 4010416).

## Disclosure

All authors have read and approved the final version of the manuscript. The corresponding author had full access to all of the data in this study and takes complete responsibility for the integrity of the data and the accuracy of the data analysis.

## Ethics Statement

Approval for this study was obtained from the Ethics Committee of Mashhad University of Medical Sciences, School of Dentistry, under Project Numbers 4010376 and 4010416 (Ethics Approval Codes IR.MUMS.DENTISTRY.REC.1401.05 and IR.MUMS.DENTISTRY.REC.1401.058).

## Consent

The authors have nothing to report.

## Conflicts of Interest

The authors declare no conflicts of interest.

## Supporting Information

Additional supporting information can be found online in the Supporting Information section.

## Supporting information


**Supporting Information** Table S1: The Checklist for Reporting In‐vitro Studies (CRIS).

## Data Availability

The data supporting the findings of this study are available within the article and its Supporting Information. Additional data are available from the corresponding author upon reasonable request.
