# Supplementary material for: Impact of Tooth Preparation Techniques on Fracture Strength of Composite‐Restored Teeth: A Focus on Deep Margin Elevation With Sandblasting and EDTA
Source: Int J Dent. 2026 Nov 3;2026:5514582. doi: 10.1155/ijod/5514582 (PMC13731979; doi:10.1155/ijod/5514582)
Supplement: Supplementary file 1 [file IJOD-2026-5514582-s001.docx]

| Supplementary table 1: The Checklist for Reporting In-vitro Studies (CRIS) | | | |
| --- | --- | --- | --- |
| Item | **CRIS reporting item** | **Reported** | **Location in manuscript** |
| 1 | Title identifies the study as an in vitro investigation | Yes | Page 1 |
| 2 | Structured abstract | Yes | Page 1–2 |
| 3 | Scientific background and rationale | Yes | Pages 3–5 |
| 4 | Study objectives/hypothesis | Yes | Page 5 |
| 5 | Study design identified | Yes | Page 6 |
| 6 | Ethical approval | Yes | Pages 6, 19 |
| 7 | Specimen source and eligibility criteria | Yes | Pages 6–7 |
| 8 | Sample size justification | Yes | Page 6 |
| 9 | Randomization method | Yes | Page 8 |
| 10 | Allocation to experimental groups | Yes | Pages 7–8 |
| 11 | Blinding | Yes | Page 12 |
| 12 | Intervention details sufficient for replication | Yes | Pages 7–11 |
| 13 | Materials and manufacturers reported | Yes | Pages 7–11 |
| 14 | Outcome measures clearly defined | Yes | Pages 6, 12 |
| 15 | Statistical methods | Yes | Page 12 |
| 16 | Numbers analyzed | Yes | Page 13 |
| 17 | Descriptive statistics | Yes | Pages 13, 27 |
| 18 | Main statistical results | Yes | Pages 13–14 |
| 19 | Secondary outcomes reported | Yes | Pages 13–14 |
| 20 | Interpretation of findings | Yes | Pages 14–18 |
| 21 | Comparison with previous literature | Yes | Pages 15–17 |
| 22 | Strengths and limitations | Yes | Pages 17–18 |
| 23 | Conclusions consistent with results | Yes | Page 19 |
| 24 | Funding, conflicts of interest, author contributions | Yes | Pages 19–20 |
| 25 | Availability of data | Yes | Page 19 |
